# Local and Systemic CD4^+^ T Cell Exhaustion Reverses with Clinical Resolution of Pulmonary Sarcoidosis

**DOI:** 10.1155/2017/3642832

**Published:** 2017-11-06

**Authors:** Charlene Hawkins, Guzel Shaginurova, D. Auriel Shelton, Jose D. Herazo-Maya, Kyra A. Oswald-Richter, Joseph E. Rotsinger, Anjuli Young, Lindsay J. Celada, Naftali Kaminski, Carla Sevin, Wonder P. Drake

**Affiliations:** ^1^Division of Infectious Diseases, Department of Medicine, Vanderbilt University School of Medicine, Nashville, TN 37232-2363, USA; ^2^Department of Pathology, Microbiology and Immunology, Vanderbilt University School of Medicine, Nashville, TN 37232-2363, USA; ^3^Section of Pulmonary, Critical Care and Sleep Medicine, Yale School of Medicine, New Haven, CT 06520, USA; ^4^Division of Allergy, Pulmonary and Critical Care Medicine, Department of Medicine, Vanderbilt University School of Medicine, Nashville, TN 37232-2363, USA

## Abstract

Investigation of the Th1 immune response in sarcoidosis CD4^+^ T cells has revealed reduced proliferative capacity and cytokine expression upon TCR stimulation. In other disease models, such cellular dysfunction has been associated with a step-wise, progressive loss of T cell function that results from chronic antigenic stimulation. T cell exhaustion is defined by decreased cytokine production upon TCR activation, decreased proliferation, increased expression of inhibitory cell surface receptors, and increased susceptibility to apoptosis. We characterized sarcoidosis CD4^+^ T cell immune function in systemic and local environments among subjects undergoing disease progression compared to those experiencing disease resolution. Spontaneous and TCR-stimulated Th1 cytokine expression and proliferation assays were performed in 53 sarcoidosis subjects and 30 healthy controls. PD-1 expression and apoptosis were assessed by flow cytometry. Compared to healthy controls, sarcoidosis CD4^+^ T cells demonstrated reductions in Th1 cytokine expression, proliferative capacity (*p* < 0.05), enhanced apoptosis (*p* < 0.01), and increased PD-1 expression (*p* < 0.001). BAL-derived CD4^+^ T cells also demonstrated multiple facets of T cell exhaustion (*p* < 0.05). Reversal of CD4^+^ T cell exhaustion was observed in subjects undergoing spontaneous resolution (*p* < 0.05). Sarcoidosis CD4^+^ T cells exhibit loss of cellular function during progressive disease that follows the archetype of T cell exhaustion.

## 1. Introduction

Sarcoidosis is a Th1 granulomatous disease for which the incidence and mortality continue to rise [1]. Pulmonary sarcoidosis is characterized by striking clinical heterogeneity in that over half of subjects will spontaneously resolve their disease, while the remainder experience progressive loss of lung function. Although the etiology of sarcoidosis is not known, a growing body of literature demonstrates that alterations in immune function and the immunogenetic transcriptome contribute to clinical outcome. Despite spontaneous secretion of Th1 (and Th2) cytokines such as IL-2 and IFN-*γ* [2–4], sarcoidosis CD4^+^ T cells demonstrate suboptimal Th1 cytokine production and proliferation following T cell receptor (TCR) stimulation during active disease. It has also been reported that reduced proliferative capacity, upregulation of inhibitory receptors, such as programmed death 1 (PD-1), and B cell dysfunction are present in cells derived from sarcoidosis patients experiencing disease progression [5–9]. Both immune dysfunction and PD-1 upregulation were reversed in subjects during spontaneous clinical resolution [5], supporting the notion that immune dysfunction contributes to sarcoidosis disease progression.

The observation of reduced cytokine expression upon TCR stimulation as well as upregulation of PD-1 suggests an altered T cell differentiation state characterized by progressive and hierarchical loss of effector function, termed T cell exhaustion. Although T cell exhaustion was originally described in chronic viral infections in mice, it has also been reported in chronic inflammatory states such as HIV infection and cancer [10, 11]. Exhausted cells display reduced cytokine production and proliferation in response to TCR activation with a concomitant increase in apoptosis as well as upregulation of inhibitory immune receptors such as PD-1 [10]. PD-1 expression can be upregulated following TCR stimulation and can even persist at low levels in healthy humans [12, 13]. However, elevated PD-1 expression occurring simultaneously with loss of multiple effector functions is a hallmark of T cell exhaustion [10, 11].

Little is known regarding an extensive, longitudinal characterization of sarcoidosis CD4^+^ T cell adaptive immune function in subjects with disease progression compared to disease resolution. Furthermore, while the importance of T cell exhaustion has been defined in tumor immunity [14], its relevance in interstitial lung diseases, such as sarcoidosis, has not been delineated. Here, we characterize systemic and local CD4^+^ T cell immune function in pulmonary sarcoidosis subjects clinically experiencing disease progression or spontaneous resolution. This work demonstrates that sarcoidosis CD4^+^ T cells display an exhausted phenotype during progressive disease that is reversed among subjects experiencing disease resolution. Furthermore, CD4^+^ T cells derived from local environments exhibit greater immune dysfunction than systemic CD4^+^ T cells. The reversal of the T cell exhaustion immunophenotype with spontaneous clinical resolution suggests that adaptive immune function plays an important role in sarcoidosis pathogenesis. Further in vivo studies to determine if CD4^+^ T cell exhaustion is causal of sarcoidosis disease progression is warranted.

## 2. Methods

### 2.1. Subject Characterization

For inclusion in this study, the clinical, histologic, and microbiologic criteria used to define sarcoidosis were as previously described [15]. All subjects provided written informed consent that was approved by the appropriate Institutional Review Boards. Sarcoidosis patients with progressive disease were defined as the following: (1) decline in FVC, (2) physician consideration of dose escalation of immunosuppressive therapy to treat disease-associated symptoms, and/or (3) appearance of extrapulmonary disease. Peripheral blood samples for all experiments were obtained during evidence of disease progression or clinical resolution. Subjects who experienced a decline in forced vital capacity (FVC) or with resolving disease (normalized FVC) who agreed to a research bronchoscopy were enrolled. Study participant demographics for peripheral blood mononuclear cells (PBMC) and bronchoalveolar lavage (BAL) are included in Tables 1 and 2. We noted no distinctions in immune function based upon whether patients were on immunosuppressive therapy or not (Supplemental Table 1 available online at https://doi.org/10.1155/2017/3642832). Approximately one-third of the study subjects had participated in a prior investigation [5, 8].

### 2.2. Cell Isolation and Culture

Peripheral blood and BAL fluid were processed as previously described [5, 16]. CD4^+^ T cells were purified from fresh or cryopreserved PBMC by magnetic separation (Dynal CD4 Positive Isolation Kit; Invitrogen, Carlsbad, CA). Resting CD4^+^ T cells were activated by cross-linking with plate-bound anti-CD3 (OKT-3; American Type Culture Collection) and soluble anti-CD28 (1 *μ*g/mL; BD Biosciences, San Jose, CA) as previously described [17]. CD4^+^ T cells were cultured and maintained in RPMI 1640 supplemented with 10% fetal bovine serum and 1.2% each of Penicillin Streptomycin and L-Glutamine, along with MEM vitamins, amino acids, and sodium pyruvate (all from Mediatech, INC, Manassas, VA). PBMC viability of >95% was confirmed by trypan blue in all analyses.

### 2.3. Cytokine Production and Proliferation Assays

Cytokine bead array (BD Biosciences, San Jose, CA) was conducted to assay spontaneous and TCR-stimulated cytokine secretion as previously described [17]. Flow cytometry analysis was performed using a FACSCalibur flow cytometer, and flow cytometry data were analyzed using FCAP Array software. Intracellular staining and proliferation assays were conducted as previously described [5, 17]. Proliferation assays were the last to be conducted because they required the largest number of cells. Thus, proliferation analysis was limited by cellular availability.

### 2.4. Flow Cytometry

CD4^+^ T cells were stained with the relevant antibodies on ice for 30 minutes prior to washing and fixation. PD-1 expression and cellular apoptosis were measured with LSRFortessa and 3 Laser LSR-II flow cytometers, respectively. A minimum of 30,000 events were acquired per sample. Analysis was performed using FlowJo v10 software.

### 2.5. Apoptosis

Cryopreserved PBMC were thawed and sorted magnetically for CD4^+^ T cells that were collected and immediately stained using PE-conjugated Annexin V (BD Pharmingen, San Diego, CA). Flow cytometry was used to analyze cells for 7AAD and Annexin V expression. For matching BAL and PBMC analysis, BAL cells and PBMC were thawed and stained with anti-CD3 and anti-CD4 and washed twice prior to staining for expression of 7AAD and Annexin V.

### 2.6. PD-1 Staining

Fresh or cryopreserved PBMC or BAL was washed with FACS Buffer and incubated with anti-CD3, anti-CD4, anti-CD8, anti-CD45RO, anti-CCR7, and anti-PD-1 (all antibodies obtained from BD Pharmingen, San Diego, CA) for 30 minutes before fixation and flow cytometry. Using FlowJo software, live, singlet lymphocyte populations were gated on forward- and side-scatter properties before cells were analyzed for CD3, CD4, and CD8 expression. The naïve population was isolated by gating CD3^+^CD4^+^ cells for CCR7^hi^ and CD45RO^lo^ expression. PD-1 expression was determined in the naïve population, and this gate was dropped on the total CD3^+^CD4^+^ population to delineate PD-1 expression in the total population of CD4^+^ T cells.

### 2.7. Genetic Analyses

The microarray gene expression dataset, GSE1907, was downloaded from the National Center for Biotechnology Information's Gene Expression Omnibus (GEO). Data were analyzed using significant analysis of microarrays, as previously described [5]. A stringent false-discovery rate cutoff of less than 1% was used to define statistical significance, using PCluster to depict microarray analysis results. Quantitative RT-PCR analysis of sarcoidosis CD4^+^ T cells was performed as previously described [8].

### 2.8. Statistical Analysis

Data reported as the arithmetic mean ± SD. Comparisons between cohorts were performed using an unpaired two-tailed Student *t*-test. Multiple group comparisons were performed using a one-way analysis of variance (ANOVA) with Tukey's honest significant difference (HSD). Proliferation data were analyzed using the Mann–Whitney *U* test. Statistical analysis for all figures was performed using Prism version 6.0 (GraphPad software). A *p* value < 0.05 was considered statistically significant. All data was used in analysis.

## 3. Results

### 3.1. Sarcoidosis CD4^+^ T Cells Display Spontaneous Cytokine Expression but Reduced Cytokine Secretion after TCR Stimulation

**Figure 1:**
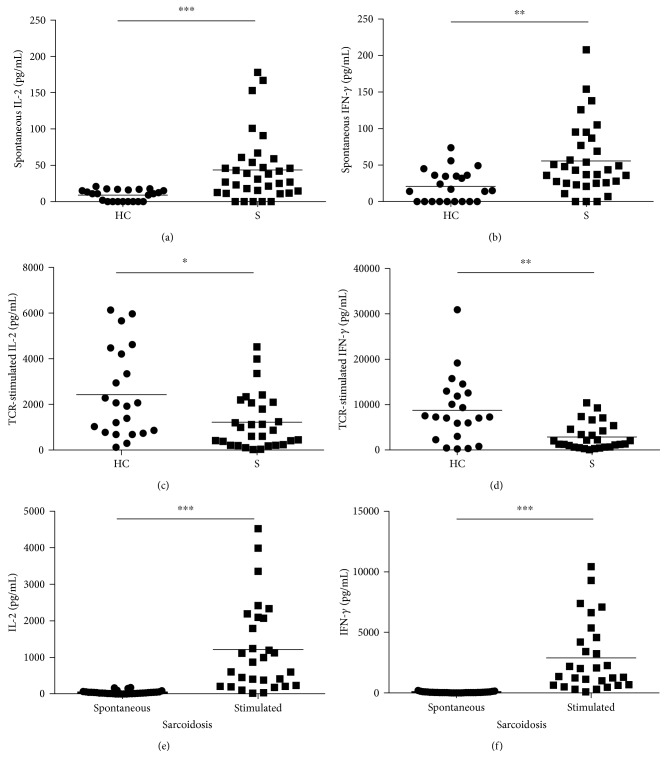
Spontaneous and TCR-stimulated cytokine secretion in CD4^+^ T cells. CD4^+^ T cells were isolated from the PBMC of healthy control (HC) or sarcoidosis (S) subjects and cultured for 24 hours in the absence (a and b) or presence (c and d) of polyclonal TCR stimulation. Cell culture supernatants were collected 24 hours post TCR stimulation and analyzed for cytokine secretion by cytokine bead array. (a) Spontaneous IL-2 secretion in pg/mL. (b) Spontaneous IFN-*γ* secretion in pg/mL. (c) TCR-stimulated IL-2 secretion in pg/mL. (d) TCR-stimulated IFN-*γ* secretion in pg/mL. (e) Spontaneous and TCR-stimulated IL-2 secretion by sarcoidosis CD4^+^ T cells in pg/mL. (f) Spontaneous and TCR-stimulated IFN-*γ* secretion by sarcoidosis CD4^+^ T cells in pg/mL. Horizontal lines represent mean values of cytokine secretion for each cohort. Statistical analysis was performed using the Mann–Whitney *U* test. ^∗^*p* < 0.05, ^∗∗^*p* < 0.01, and ^∗∗∗^*p* < 0.001.

A central immunologic feature of CD4^+^ T cells in sarcoidosis is the spontaneous release of Th1 and Th2 cytokines [2–4]—a phenotype indicative of active disease status in pulmonary sarcoidosis at both peripheral and local inflammation sites in the lungs [8, 18]. When we assessed for spontaneous Th1 cytokine secretion, on average, CD4^+^ T cells purified from sarcoidosis PBMC secreted nearly 5-fold higher levels of IL-2 (Figure 1(a); 43.6 pg/mL versus 8.97 pg/mL, *p* < 0.0001) and more than 2-fold higher levels of IFN-*γ* (Figure 1(b); 55.8 pg/mL versus 20.95 pg/mL, *p* < 0.01) than those purified from healthy controls, findings consistent with previous investigations [18].

Despite evidence of higher spontaneous Th1 cytokine expression in sarcoidosis CD4^+^ T cells, following TCR stimulation, systemic CD4^+^ T cells isolated from healthy controls produced significantly higher amounts of IL-2 than sarcoidosis CD4^+^ T cells, with an average of 2431 pg/mL compared to an average of 1220 pg/mL, respectively (Figure 1(c); *p* < 0.05). A similar phenotype was observed when IFN-*γ* secretion was assessed after TCR stimulation: on average, healthy control cells secreted 8757 pg/mL of IFN-*γ* versus 2897 pg/mL of IFN-*γ* from sarcoidosis cells (Figure 1(d); *p* < 0.01).

To more carefully assess the capacity of sarcoidosis CD4^+^ T cells to produce cytokines, we compared spontaneous versus TCR-stimulated IL-2 and IFN-*γ* production (Figures 1(e) and 1(f)). This analysis revealed that nearly 30-fold IL-2 and 5-fold IFN-*γ* were produced upon TCR stimulation above the level of spontaneous secretion of each cytokine (Figures 1(e) and 1(f); *p* < 0.0001), demonstrating that while sarcoidosis CD4^+^ T cells were exhibiting spontaneous Th1 cytokine expression, the amount secreted was not at capacity.

### 3.2. Sarcoidosis CD4^+^ T Cells Exhibit Reduced Proliferation upon TCR Stimulation

**Figure 2:**
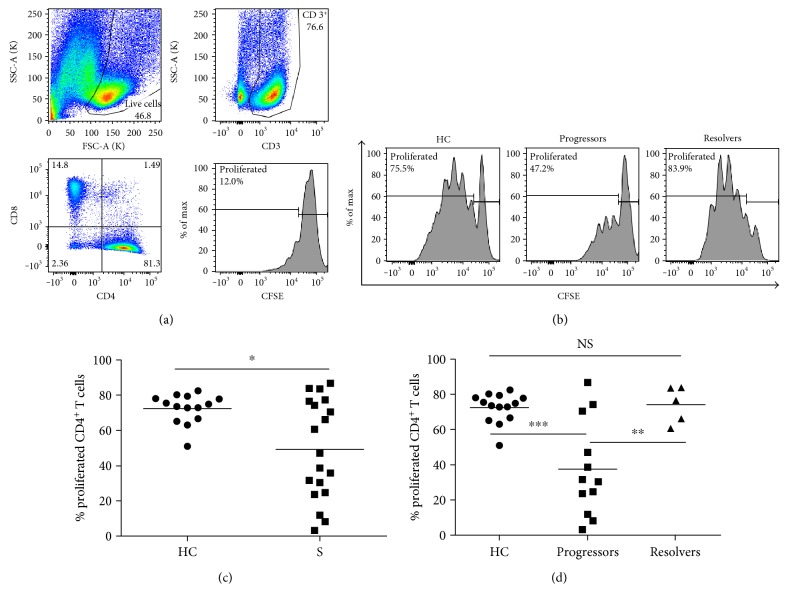
Proliferative capacity in sarcoidosis CD4^+^ T cells. PBMC were labeled with 2.5 *μ*M CSFE, TCR stimulated, and cultured for 5 days in supplemented RPMI 1640 medium. Cells that had not received polyclonal TCR activation were cultured alongside the stimulated cells as a negative control. On day 5, cells were collected and stained with anti-CD3, anti-CD4, and anti-CD8. After fixation, cells were analyzed by flow cytometry to assess cell size and the intensity of the CFSE signal. (a) Representative flow cytometry data illustrating the gating strategy used to quantitate the percentage of proliferating cells. Briefly, live, singlet CD3^+^ cells were gated according to CD4 and CD8 expression. The population of CD4^+^ cells was then gated based on the intensity of CFSE signal. The peak at the far right on the CFSE histogram represents cells that had not proliferated by day five; peaks to the left of this nonproliferating population indicate cells that had proliferated. (b) Representative histograms of sarcoidosis progressor and resolver proliferating CD4^+^ T cells. (c) Plot of healthy control (HC) and sarcoidosis proliferating CD4^+^ T cells. Statistical analysis was performed using the Mann–Whitney *U* test. (d) Plot of healthy control and sarcoidosis progressor and resolver proliferating CD4^+^ T cells. Statistical analysis was performed using one-way ANOVA with Tukey's HSD. Horizontal lines represent mean percentages of proliferating CD4^+^ T cells. ^∗^*p* < 0.05, ^∗∗^*p* < 0.01, and ^∗∗∗^*p* < 0.001. NS: not significant.

In healthy CD4^+^ T cells, a robust adaptive immune response upon challenge with antigen involves cellular proliferation [19]. However, exhausted T cells exhibit reduced proliferation upon TCR stimulation [20, 21]. We sought to examine how systemic sarcoidosis T cells proliferate after polyclonal TCR stimulation. We noted that when the percentage of TCR activated, proliferating sarcoidosis CD4^+^ cells were measured, only an average of 49.3% of cells proliferated whereas an average of 72.5% of healthy control CD4^+^ T cells proliferated after TCR stimulation (Figures 2(a), 2(b), and 2(c); *p* < 0.05).

The sarcoidosis cohort displayed a range of proliferation capacities: those with proliferation akin to healthy control populations and those with lower proliferative capacities (Figure 2(c)). We assessed the clinical status of each subject within the sarcoidosis cohort, separating subjects into two populations based on clinical criteria: those with active, progressive disease (progressors) and those experiencing disease resolution (resolvers). With an average of 36.6% proliferated cells, this analysis revealed that sarcoidosis progressors had proliferation values significantly lower than that of healthy controls and sarcoidosis resolvers (Figure 2(d); *p* < 0.001 and *p* < 0.01, resp.). However, sarcoidosis resolvers demonstrated proliferative capacities comparable to that of the healthy control cohort (*p* = 1.0).

### 3.3. Sarcoidosis CD4^+^ T Cells Exhibit Increased Levels of Apoptosis

**Figure 3:**
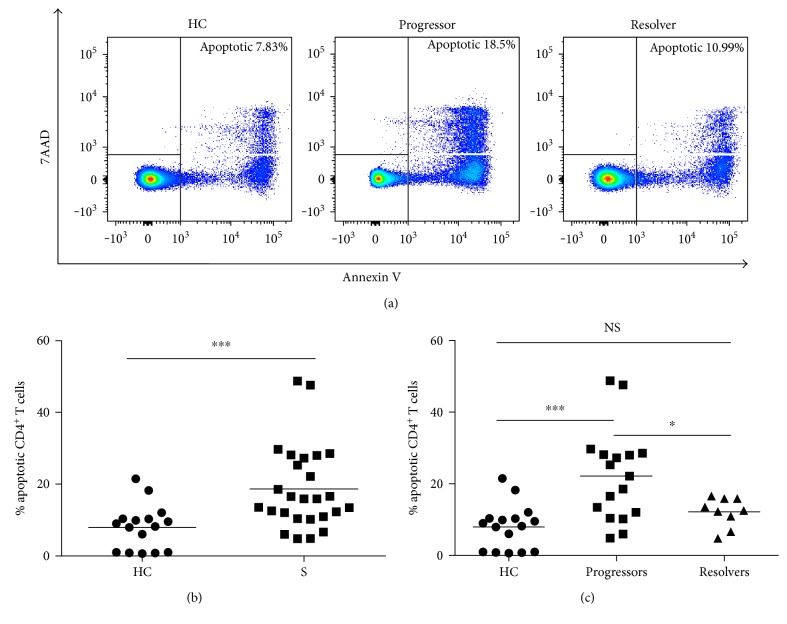
Increased spontaneous apoptosis in sarcoidosis CD4^+^ T cells. CD4^+^ T cells were sorted from healthy control (HC) and sarcoidosis (S) PBMC and stained at baseline with apoptosis biomarkers, Annexin V and 7AAD. Flow cytometry was conducted, and FlowJo software was used to gate on apoptotic cells. (a) Representative flow cytometry histograms illustrating the gating strategy used to determine the population of apoptotic cells. (b) Plot of healthy control and sarcoidosis apoptosing CD4^+^ T cells. Statistical analysis was performed using the Mann–Whitney *U* test. (c) Plot of healthy control versus sarcoidosis progressor and resolver apoptosing CD4^+^ T cells. Statistical analysis was performed using one-way ANOVA with Tukey's HSD. Horizontal lines represent mean percentages of apoptosing CD4^+^ T cells. ^∗^*p* < 0.05 and ^∗∗∗^*p* < 0.001. NS: not significant.

The most exhausted cells possess the highest levels of susceptibility to apoptosis, a later phase in the progressive loss of function that defines T cell exhaustion [10, 20–23]. We sought to determine whether sarcoidosis CD4^+^ T cells also displayed increased levels of apoptosis at baseline by using Annexin V and 7AAD cell-surface staining. The comparison of the sarcoidosis and healthy control cohorts revealed an average of 18.7% and 7.9%, respectively, of apoptosing CD4^+^ T cells (Figures 3(a) and 3(b); *p* < 0.001).

Similar to the proliferation analysis of the sarcoidosis cohort, we analyzed subjects by clinical outcome, which revealed that sarcoidosis progressors had, on average, significantly higher levels of baseline apoptosis than healthy controls and sarcoidosis resolvers (Figure 3(c); *p* < 0.01 and *p* < 0.05, resp.). However, the sarcoidosis resolvers had apoptosis levels that were comparable to those of healthy controls (Figure 3(c); *p* = 0.53).

### 3.4. Increased PD-1-Expressing CD4^+^ T Cells during Active Sarcoidosis

**Figure 4:**
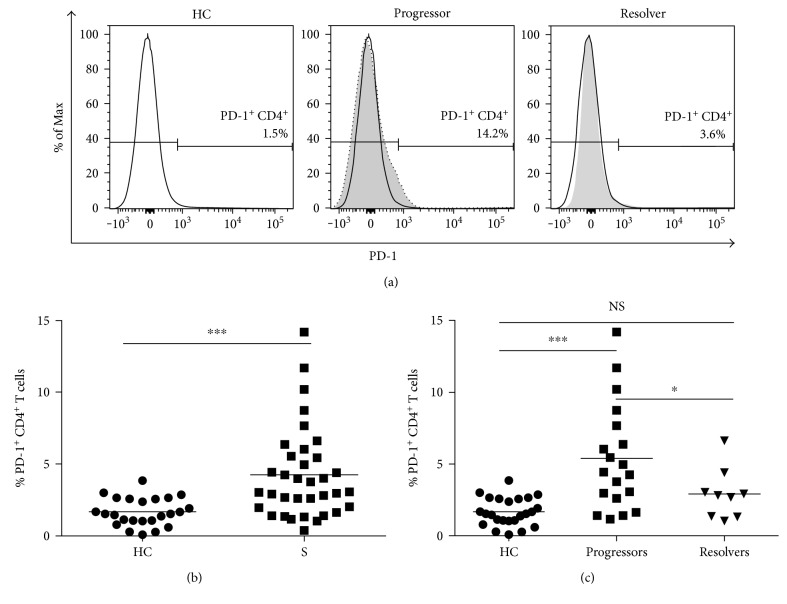
PD-1 upregulation in sarcoidosis CD4^+^ T cells. PBMC isolated from the whole blood of healthy control, and sarcoidosis subjects were stained using anti-CD3, anti-CD4, anti-CCR7, anti-CD45RO, and anti-PD-1 antibodies. (a) Representative flow cytometry histograms to illustrate PD-1 gating in the population of CD4^+^ T cells from healthy control and sarcoidosis progressor and resolver subjects. For comparison, the healthy control histogram (black trace) was overlayed with the sarcoidosis progressor and resolver histogorams. (b) Plot of the percentages of sarcoidosis PD-1^+^CD4^+^ T cells relative to healthy controls. Statistical analysis was performed using the Mann–Whitney *U* test. (c) Plot of PD-1^+^CD4^+^ T cells present during sarcoidosis disease progression and resolution relative to healthy controls. Statistical analysis was performed using one-way ANOVA with Tukey's HSD. Horizontal lines indicate mean percentages of PD-1^+^CD4^+^ T cells. ^∗^*p* < 0.05 and ^∗∗∗^*p* < 0.001. NS: not significant.

For a number of diseases, increased expression of inhibitory receptors on the surface of systemic effector cells has been associated with disease progression [10, 24–26]. Programmed death 1 (PD-1) is one such inhibitory molecule that becomes upregulated during a number of chronic disease states [24, 26, 27]. PD-1 upregulation has been shown to be a significant factor contributing to the loss of cytokine expression, proliferative capacity, and increased susceptibility to apoptosis observed during T cell exhaustion [27–30]. Investigation of PD-1 expression in sarcoidosis and healthy control cohorts revealed an average of 4.2% PD-1^+^CD4^+^ sarcoidosis T cells while the average percentage of PD-1^+^CD4^+^ T cells in the healthy control cohort was approximately 1.6% (Figures 4(a) and 4(b); *p* < 0.0001).

Similar to previous analyses in the sarcoidosis cohort (Figures 2 and 3), we also subdivided this sarcoidosis cohort based upon disease state. The average percentage of PD-1^+^CD4^+^ T cells was significantly higher in sarcoidosis progressors than in healthy controls and sarcoidosis resolvers (Figure 4(c); *p* < 0.0001 and *p* < 0.05, resp.). However, there was no significant difference between the average percentages of PD-1^+^CD4^+^ cells in sarcoidosis resolvers versus healthy controls (Figure 4(c); *p* = 0.4).

### 3.5. Sarcoidosis Pathogenesis Involves Multiple Facets of Immune Dysfunction Not Present during Disease Resolution

**Figure 5:**
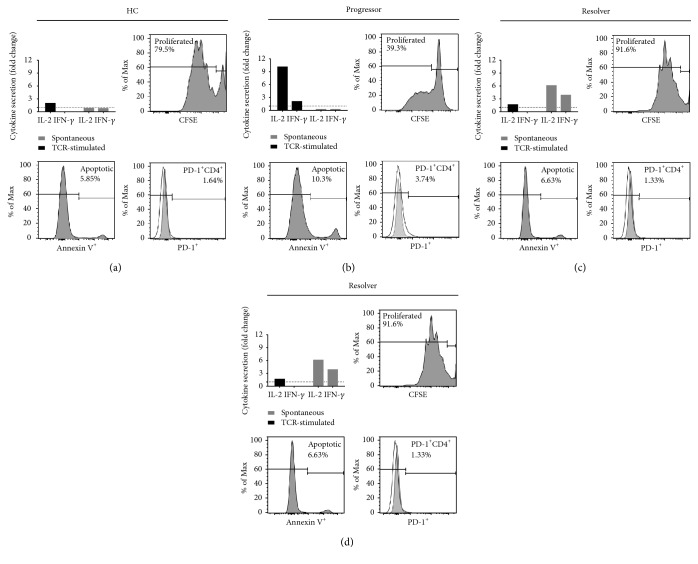
CD4^+^ T cells from sarcoidosis progressors exhibit characteristics of exhaustion. (a, b, and c) Representative functional data for a single healthy control, sarcoidosis progressor, and sarcoidosis resolver. (a) Cytokine secretion; data have been normalized to the mean value for the healthy control cohort (hashed line). (b and c) Proliferation analysis. (a) Apoptosis analysis. (b and c) Percent PD-1^+^CD4^+^ T cells; overlay of naïve (CCR7^+^CD45RO^−^; shaded) CD4^+^ cells and total CD4^+^ T cells (unshaded). (d) Immune activity of sarcoidosis progressors (*n* = 12) and resolvers (*n* = 5) relative to healthy controls. High = activity greater than one standard deviation *above* the healthy control mean (yellow). Low = activity greater than one standard deviation *below* the healthy control mean (magenta). Normal = *within* one standard deviation of the healthy control mean (grey). White spaces indicate data not present due to lack of available cells.

As a cohort, sarcoidosis CD4^+^ T cells demonstrated reduced cytokine expression and proliferative capacity upon TCR stimulation as well as upregulated PD-1 and a higher percentage of apoptotic cells at baseline (Figures 1–4). T cell exhaustion is characterized by the presence of multiple defects in adaptive immunity in a single population of cells. The immunophenotype of sarcoidosis CD4^+^ T cells was examined, conducting analysis of spontaneous and TCR-stimulated cytokine expression, proliferation, apoptosis, and PD-1 expression for each sarcoidosis subject until the limit of PBMC availability was reached. In Figures 5(a), 5(b), and 5(c), the functional data for representative healthy control and sarcoidosis progressor and resolver subjects are presented.

We sought to categorically assess the level to which each immune property associated with exhausted T cells is also observed in individual sarcoidosis subjects. For each immune parameter examined, the mean value exhibited by the healthy control cohort served as the standard. The relative activity of each property is presented on a scale from high (yellow), representing more than one standard deviation above the healthy control mean, to low (magenta), representing greater than 1 standard deviation below the mean of healthy controls (Figure 5(b)). The normal designation (grey) is within one standard deviation of the healthy control mean.

We also subdivided the sarcoidosis cohort according to clinical outcome. Sarcoidosis progressors typically demonstrated multiple immune defects, including increased spontaneous cytokine production and reduced TCR-stimulated Th1 cytokine expression and proliferation. Increased percentages of apoptotic cells and PD-1 expression were also noted (Figure 5(b)). Conversely, sarcoidosis resolvers demonstrated multiple facets of immune recovery: decreased spontaneous cytokine expression along with TCR-stimulated Th1 cytokine levels and proliferative capacity akin to the healthy control cohort. Among the resolvers, PD-1 expression as well as the percentage of apoptotic cells remained elevated for some subjects; however, some resolvers demonstrated normal apoptosis and PD-1 levels (Figure 5(b)).

### 3.6. Sarcoidosis BAL-Derived CD4^+^ T Cells Exhibit Multiple Facets of Immune Dysfunction

**Figure 6:**
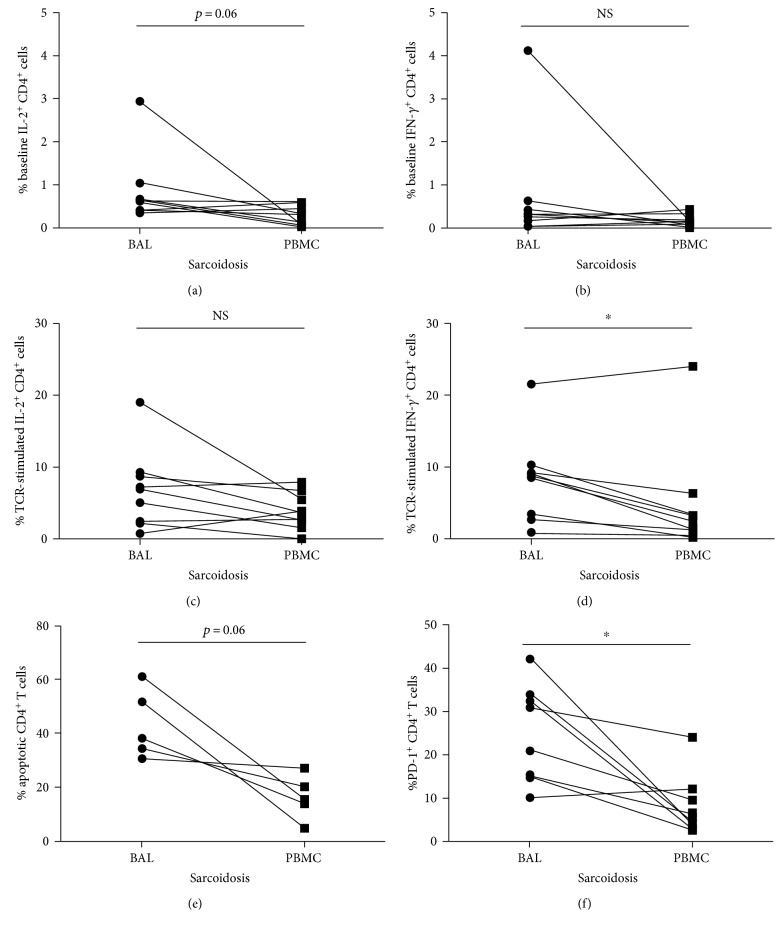
Immune dysfunction in sarcoidosis BAL versus PBMC. BAL cells and matching PBMC isolated from sarcoidosis subjects were analyzed according to immune parameters associated with T cell exhaustion. (a, b, c, and d) Intracellular staining for cytokine production: cells were incubated in the absence (a and b) or presence (c and d) of TCR stimulation for 6 hours with Golgi Stop. Cells were then stained with anti-CD3, anti-CD4, and anti-CD8 before permeabilization and staining with PE-conjugated anti-IL-2. Flow cytometry was conducted and live, singlet cells were gated to assess the percentage of CD4^+^CD3^+^ cells that had produced IL-2 or IFN-*γ*. Unstimulated cells (baseline) were used to help determine the IL-2 production in TCR-stimulated cells. (a and b) Baseline IL-2 and IFN-*γ* production. (c and d) TCR-stimulated IL-2 and IFN-*γ* production. (e) The percentage of apoptosing CD4^+^ T cells in matching BAL and PBMC was assessed using 7AAD and Annexin V staining. (f) PD-1 expression in the population of CD4^+^ T cells was determined in matching BAL and PBMC. Statistical analysis was performed using the Wilcoxon matched-pairs signed-rank test. ^∗^*p* < 0.05. NS: not significant.

Because >90% of sarcoidosis subjects experience pulmonary involvement [31, 32], we sought to characterize cytokine production, apoptosis, and PD-1 expression among bronchoalveolar lavage- (BAL-) derived sarcoidosis CD4^+^ T cells. Proliferative capacity was not assessed due to limitations in cellular availability. Intracellular staining for IL-2 and IFN-*γ* production in matching sarcoidosis BAL and PBMC was conducted at baseline and after TCR stimulation. The percentages of IL-2-producing CD4^+^ T cells in BAL relative to the periphery at baseline approached significance (Figure 6(a), *p* = 0.06), while no distinctions in the percentage of or IFN-*γ*-producing CD4^+^ T cells were noted (*p* = 0.25). After TCR stimulation, intracellular staining for IL-2 production in matching BAL and PBMC revealed no significant difference in the percentage of cells producing IL-2 (Figure 6(c); *p* = 0.1). However, a significantly greater percentage of BAL-derived CD4^+^ cells produced IFN-*γ* following TCR stimulation than those found in PBMC (Figure 6(d); *p* < 0.05). Annexin V and 7-AAD staining was conducted in matching sarcoidosis BAL and PBMC to ascertain the percentage of apoptotic cells. These data revealed an average of 43.1% and 16.3% apoptosing CD4^+^ cells in BAL and PBMC, respectively (Figure 6(e); *p* = 0.06). Finally, examination of PD-1 expression in matching sarcoidosis BAL and PBMC demonstrated significantly more PD-1^+^CD4^+^ cells in BAL than in PBMC, in which on average 25.1% versus 8.4% PD-1^+^CD4^+^ cells are present, respectively (Figure 6(f); *p* < 0.05). We have shown that sarcoidosis CD4^+^ T cells derived from the periphery exhibit immune dysfunction when compared to healthy control cells (Figures 1–4). Together, these data reveal that the phenotypes observed in the periphery also occur at the site of local inflammation in the lung, a phenomenon also observed in sarcoidosis innate immune cells [33]. Thus, multiple immune defects characteristic of T cell exhaustion are present in sarcoidosis BAL.

### 3.7. Longitudinal Analysis Reveals That T Cell Exhaustion Is Reversed during Disease Resolution

**Figure 7:**
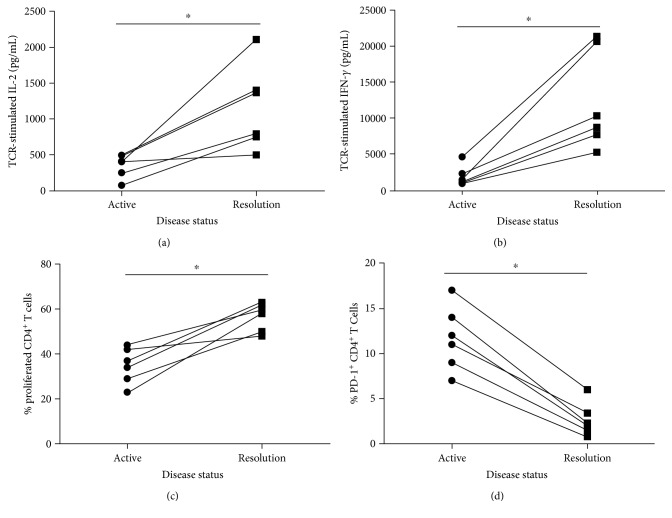
Longitudinal data of sarcoidosis immune function during active disease versus resolution. CD4^+^ T cells from sarcoidosis subjects that had resolved their disease while participating in our study were analyzed for parameters associated with T cell exhaustion during active disease and during disease resolution. Statistical analysis was performed using the Wilcoxon matched-pairs signed-rank test. ^∗^*p* < 0.05. (a) TCR-stimulated IL-2 secretion in pg/mL. (b) TCR-stimulated IFN-*γ* secretion in pg/mL. (c) Percent proliferated CD4^+^ T cells. (d) Percent PD-1^+^CD4^+^ T cells.

Sarcoidosis subjects possess multiple facets of immune dysfunction characteristic of exhausted cells (Figure 5). We conducted longitudinal analysis on PMBC obtained from the time of diagnosis through clinical resolution, comparing Th1 cytokine production and proliferation after TCR stimulation, and baseline PD-1 expression for each subject in this subset. In each case, we observed that during disease resolution, there was a significant increase in TCR-stimulated IL-2 and IFN-*γ* secretion as compared to the level of cytokine secreted during active disease (Figures 7(a) and 7(b); *p* < 0.05). We also observed an increase in the percentage of CD4^+^ T cells that proliferated in response to TCR stimulation during disease resolution (Figure 7(c); *p* < 0.05). The percentage of PD-1^+^CD4^+^ cells had also significantly decreased during disease resolution as compared to during active disease (Figure 7(d); *p* < 0.05). These data indicate that while individuals with active sarcoidosis exhibit characteristics of exhausted T cells, upon disease resolution, the exhaustion phenotype is reversed concomitant with the acquisition of normal function in several immune parameters.

### 3.8. Correlation between *BATF* and PD-1 Expression in Sarcoidosis CD4^+^ T Cells

**Figure 8:**
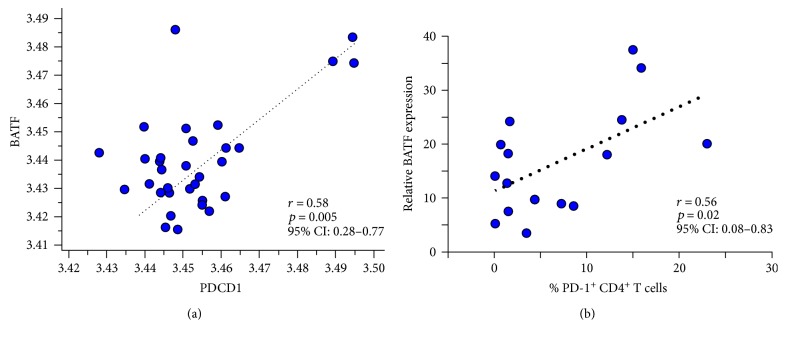
Positive correlation between *BATF* and PD-1 in sarcoidosis PBMC. (a) Correlation between log_2_-transformed microarray expression values of BATF and *PDCD1* in sarcoidosis PBMC (*n* = 32). *BATF* expression is positively correlated with *PDCD1* expression in PBMC, indicating a statistically significant association between these two variables. (b) Correlation between quantitative RT-PCR values of *BATF* transcript and PD-1 expression in sarcoidosis CD4^+^ T cells (*n* = 16). *BATF* expression is positively correlated with PD-1 expression in CD4^+^ T cells, indicating a statistically significant association between these two variables. Statistical analysis was performed using Student's *t*-distribution for Pearson's correlation.

We observed evidence of T cell exhaustion in sarcoidosis BAL and PBMC, a phenotype mediated, at least in part, by PD-1 upregulation in the population of CD4^+^ cells. We sought to examine molecular events related to PD-1 signaling that could contribute to the exhaustion phenotype. Microarray analysis of sarcoidosis PBMC revealed a positive correlation between the expression of *PDCD1*, the gene encoding PD-1, and basic leucine zipper transcription factor ATF-like (*BATF*) (Figure 8(a); *r* = 0.58, *p* = 0.005). *BATF* is a master regulator transcription factor that controls multiple aspects of T cell function, including cellular activation, differentiation, and proliferation [34]. Previous work has demonstrated a positive correlation between *BATF* expression and PD-1 signaling in exhausted CD8^+^ T cells [28]. Analysis of *BATF* transcript and PD-1 protein expression in sarcoidosis CD4^+^ T cells revealed a positive relationship between *BATF* and PD-1, such that sarcoidosis subjects with high PD-1 upregulation also express higher levels of *BATF* (Figure 8(b); *r* = 0.56, *p* = 0.02). These data suggest that PD-1 signaling and *BATF* expression may mediate sarcoidosis CD4^+^ T cell exhaustion.

## 4. Discussion

This is the first report of the presence of site-specific and systemic immune defects—characterized by hierarchical loss of effector function and inhibitory receptor upregulation—occurring in sarcoidosis patients experiencing disease progression with normalization of immune function occurring in subjects with spontaneous resolution. Although these cells are not entirely functionally inert, our results demonstrate defective Th1 cytokine expression and proliferation following TCR stimulation, increased percentage of apoptotic cells, and sustained PD-1 upregulation among sarcoidosis subjects experiencing clinical progression. A striking observation was that this loss of multiple aspects of effector function is present in local (BAL) and systemic environments (PBMC) (Figure 6), a phenomenon also observed in the innate immune cells of sarcoidosis subjects [33]. Therefore, the inflammatory cells present in sarcoidosis BAL also exhibit characteristics of immune exhaustion.

An encouraging finding was that the T cell exhaustion phenomenon was reversible in sarcoidosis subjects—an observation also reported in viral infections, cancer, and other granulomatous diseases [35–37]. Thus, T cell exhaustion is not a terminal fate in sarcoidosis CD4^+^ T cells. This particularly relevant finding supports further investigation of the determinants of adaptive immune recovery within sarcoidosis subjects. Some of the sarcoidosis subjects were more than two years after their initial diagnosis of pulmonary sarcoidosis, suggesting that the length of time during which one has sarcoidosis should not be a deterrent to consider recovery of the adaptive immune response. The clinical observation of improved lung function and resolution of granulomatous inflammation supports investigation to determine whether systemic recovery reflects local recovery. Our results parallel reports of other pulmonary diseases that note the important contribution of strong adaptive immunity to clinical outcome.

One of many compelling questions is “what is driving T cell exhaustion during sarcoidosis pathogenesis?” In other chronic diseases, several mediators of T cell exhaustion have been noted, including persistent antigen exposure, upregulation of inhibitory receptors, and the activity of regulatory T cells as the leading candidates. In chronic hepatitis C, reversal of T cell exhaustion was noted with spontaneous clearance of virus, demonstrating the importance of persistent antigen load on the development of T cell dysfunction [37]. It has been recently demonstrated that the *Mycobacterium tuberculosis* cell wall glycolipid, mannose-capped lipoarabinomannan (ManLAM), induced decreased IL-2 production and proliferation in CD4^+^ T cells when the cells were restimulated with mycobacterial antigen 85B. ManLAM had been previously demonstrated to induce T cell anergy through downregulation of the proximal T cell receptor signaling pathway involving the tyrosine kinase, Lck [38–40]. These data identify mycobacterial antigens as negative regulators of host cell immune function.

Interestingly, molecular analysis of sarcoidosis granulomas reveals the presence of poorly degradable microbial antigens, specifically from mycobacteria and propionibacteria [16, 41, 42]. These secreted antigens are targets of local and systemic adaptive immune responses and could certainly contribute to the development of T cell exhaustion. We have noted T regulatory cell-independent downregulation of the proximal T cell receptor pathway through Lck modulation in sarcoidosis CD4^+^ T cells [8]. These findings parallel the observations described above in *Mycobacterium tuberculosis* infection, supporting the notion that the presence of microbial antigens may also drive immune dysfunction in sarcoidosis. Further, the phenomenon of T cell exhaustion likely creates a cellular microenvironment that is relatively permissive for antigen persistence. A feedback cycle may occur such that as antigen burden increases and persists over time, the immune response becomes progressively weakened, allowing for more and more antigen along with an increasingly weaker immune response. Thus, it may be that the persistence of these antigens promotes T cell exhaustion in sarcoidosis CD4^+^ T cells. We found that the percentage PD-1^+^CD4^+^ cells found in BAL were significantly higher than those found in PBMC (Figure 6(f)). PD-1 upregulation may result from higher antigen burden that occurs at the sites of local inflammation versus the periphery.

There is utility to the host for downregulating the immune response during conditions of persistent antigenic stimulation, such as minimizing destruction of host tissues. However, a disadvantage of this loss of effector functions is decreased likelihood of successful antigen clearance. It is also possible that this local immune dysfunction hinders effective clearance of other infectious antigens such as fungi, environmental mycobacteria, and viruses, all of which have been associated with pulmonary sarcoidosis [43].

PD-1 upregulation has been demonstrated to drive loss of sarcoidosis proliferative capacity; blockade of the PD-1 pathway restored immune function [5]. In HIV-specific T cells, PD-1 upregulation inhibits T cell function through *BATF* upregulation [28]. Microarray analysis of sarcoidosis PBMC and CD4^+^ T cells revealed a positive relationship between *BATF* and PD-1 (Figure 8). In the resolver cohort, we observed subjects with normal proliferative capacity who also had elevated percentages of PD-1^+^CD4^+^ T cells and those who did not (Figure 5). These observations suggest that multiple mechanisms could be operant in the resolution of CD4^+^ T cell exhaustion in sarcoidosis.

The limitations of these analyses are those inherent to translational investigations using human subjects. There were subjects on immune suppression and those who were not. However, there was no difference in the degree of immune dysfunction or in clinical outcome due to the presence of immunosuppression (Supplemental Table 1). Genetic variability among human subjects could contribute to the development of T cell exhaustion. Investigation in subjects possessing the protective (*HLA-DRB1∗1101*) versus susceptible (*HLA-DRB1∗0301*) MHC Class II allele may further delineate the role of genetics in immune function during sarcoidosis [44–46]. When necessary to stimulate the TCR, we deliberately chose to conduct the immune function investigations with CD3 and CD28 antibodies to avoid results influenced by HLA type. More in-depth investigation of whether persistent antigen drives T cell exhaustion or whether T cell exhaustion leads to persistent antigen in sarcoidosis will not be possible with human investigations. These results while interesting do not demonstrate a causal relationship between sarcoidosis progression and T cell exhaustion. Thus, these observations bolster the importance of developing a sarcoidosis animal model.

This investigation reports a more complete characterization of sarcoidosis CD4^+^ T cell function. Immunoprofiling of local and systemic sarcoidosis CD4^+^ cells during disease progression reveals an exhausted phenotype, indicating that loss of multiple critical facets of adaptive immune function during disease progression is a key feature of sarcoidosis pathogenesis. The observation of the reversible nature of T cell exhaustion in sarcoidosis advocates the use of immunotherapy to restore sarcoidosis CD4^+^ T cell function as a viable therapeutic target. Therefore, these data suggest a paradigm shift for individuals experiencing clinical progression of pulmonary sarcoidosis: restoration of CD4^+^ T cell immune function would be a more effective treatment strategy to promote disease resolution. Further investigation into the utility of restoring CD4^+^ T cell effector function on clinical resolution is warranted.

## Supplementary Material

Table S1. Progressor/Resolver Detailed Demographics

## Figures and Tables

**Table 1:** Demographics of sarcoidosis and control PBMC populations.

	Sarcoidosis	Healthy controls
Number	54	30
Sex (female; male)	38; 16	23; 7
Age (y), median (min, max)	46.5 (25, 67)	40.5 (23, 63)
Race	22 AA; 31 C; 1 H	10 AA; 19 C; 1 H

AA: African-American; C: Caucasian; H: Hispanic.

**Table 2:** Demographics of sarcoidosis BAL population.

	Sarcoidosis BAL
Number	15
Sex (female; male)	9; 6
Age (y), median (min, max)	47 (25, 67)
Race	6 AA; 8 C; 1 H

BAL: bronchoalveolar lavage cells; AA: African-American; C: Caucasian; H: Hispanic.
