# Supplementary material for: Local and Systemic CD4+ T Cell Exhaustion Reverses with Clinical Resolution of Pulmonary Sarcoidosis
Source: J Immunol Res. 2017 Nov 6;2017:3642832. doi: 10.1155/2017/3642832 (PMC5695030; doi:10.1155/2017/3642832)
Supplement: Supplementary file 1 — Table S1. Progressor/Resolver Detailed Demographics [file 3642832.f1.docx]

Table S1

Table S1
