# Supplementary figures and images for: Local and Systemic CD4+ T Cell Exhaustion Reverses with Clinical Resolution of Pulmonary Sarcoidosis
Source: J Immunol Res. 2017 Nov 6;2017:3642832. doi: 10.1155/2017/3642832 (PMC5695030; doi:10.1155/2017/3642832)

## Slide 1
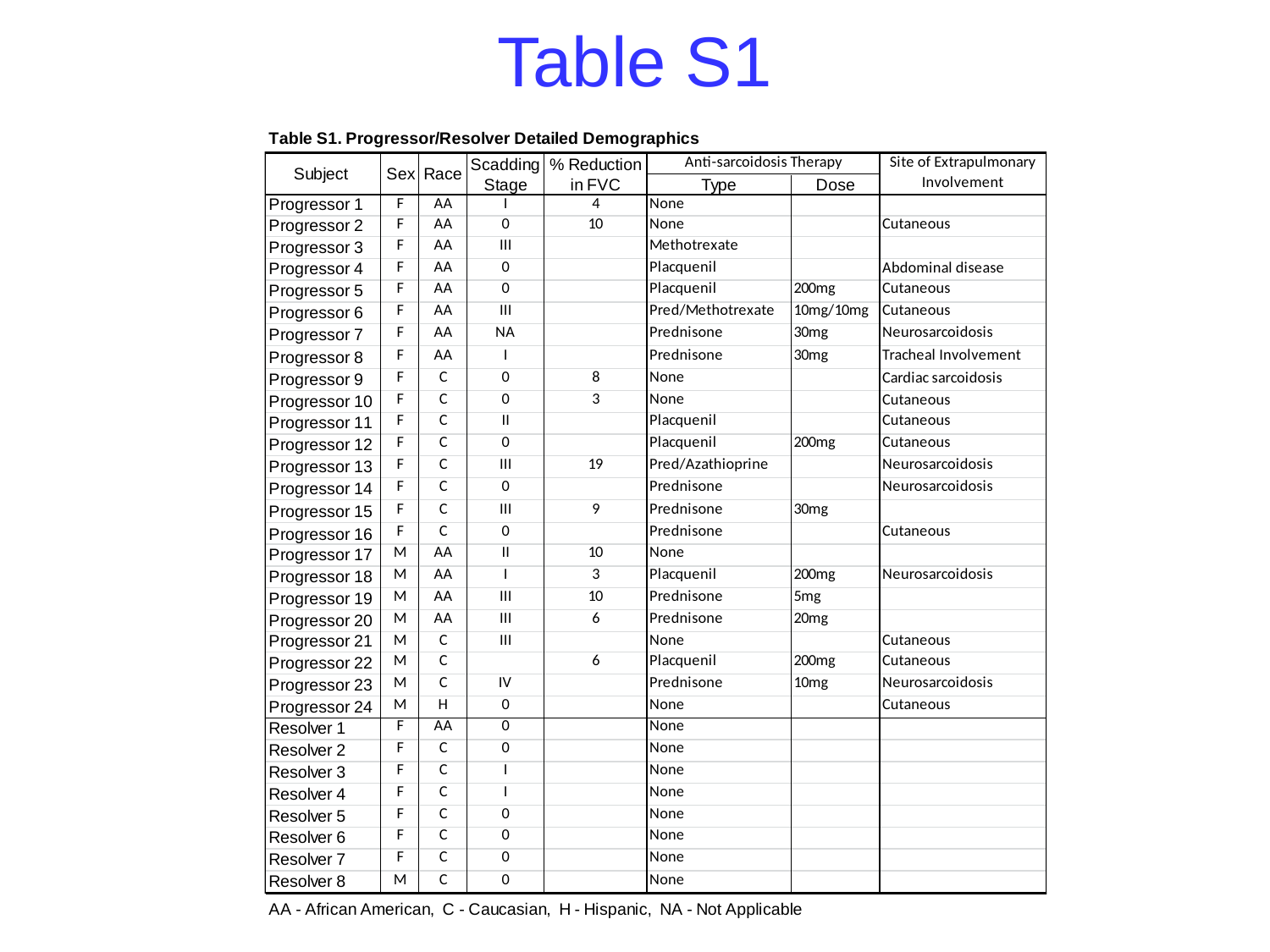

# Table S1

Supplement: Supplementary file 2 [file 3642832.f2.pptx]
